# Music Listening Predicted Improved Life Satisfaction in University Students During Early Stages of the COVID-19 Pandemic

**DOI:** 10.3389/fpsyg.2020.631033

**Published:** 2021-01-20

**Authors:** Amanda E. Krause, James Dimmock, Amanda L. Rebar, Ben Jackson

**Affiliations:** ^1^Department of Psychology, James Cook University, Townsville, QLD, Australia; ^2^Motivation of Health Behaviours Lab, School of Health, Medical and Applied Sciences, Appleton Institute, Central Queensland University, Rockhampton, QLD, Australia; ^3^School of Human Sciences (Exercise and Sport Science), University of Western Australia, Perth, WA, Australia

**Keywords:** COVID-19 pandemic, media use, leisure, music listening, life satisfaction, well-being

## Abstract

Quarantine and spatial distancing measures associated with COVID-19 resulted in substantial changes to individuals’ everyday lives. Prominent among these lifestyle changes was the way in which people interacted with media—including music listening. In this repeated assessment study, we assessed Australian university students’ media use (i.e., listening to music, playing video/computer games, watching TV/movies/streaming videos, and using social media) throughout early stages of the COVID-19 pandemic in Australia, and determined whether media use was related to changes in life satisfaction. Participants (*N* = 127) were asked to complete six online questionnaires, capturing pre- and during-pandemic experiences. The results indicated that media use varied substantially throughout the study period, and at the within-person level, life satisfaction was positively associated with music listening and negatively associated with watching TV/videos/movies. The findings highlight the potential benefits of music listening during COVID-19 and other periods of social isolation.

## Introduction

Advancements in digital technologies have substantially changed the manner in which individuals interact with media. Over the last 40 years, and especially since the advent of mobile and internet technologies, media consumption in the forms of listening to music, playing video/computer games, watching TV/movies/streaming videos, and using social media, has become a ubiquitous part of everyday life. Many youth spend more time with media than any single activity other than sleeping—the average American aged 8–18, for example, uses media for over 6 h per day (Roberts and Foehr, 2008). Researchers have provided insights into the evolving nature of media consumption, the psychological drivers of such consumption, and the effects of media consumption on various groups (e.g., Roberts and Foehr, 2008; Hoge et al., 2017). Such research is important given the prominence and ubiquity of media use in modern society; however, COVID-19 has changed the landscape of media use and its possible effects. Research is needed to highlight the changing nature of media use through stages of the COVID-19 pandemic, and reveal the extent to which media use impacted life satisfaction during these stages.

Around the world, COVID-19 quickly disrupted everyday life (Sundarasen et al., 2020; Zhang W.-R. et al., 2020; Zheng et al., 2020), causing significant physical harm for a large proportion of the community (Sibley et al., 2020). With the goal of curtailing the spread of the virus, many governments implemented strict quarantine, and spatial distancing measures. However, such measures have been associated with an increased mental health burden (Brooks et al., 2020; Fischer et al., 2020), with data obtained during COVID-19 reinforcing previous evidence that quarantine and stay-at-home orders increase anxiety, irritability, stress, insomnia, anger, uncertainty, and confusion (e.g., Bai et al., 2004; Cava et al., 2005; Brooks et al., 2020; Li et al., 2020; Sønderskov et al., 2020; Williams et al., 2020). For many, life satisfaction during COVID-19 has been negatively influenced by reduced social interactions resulting from restrictions (Ammar et al., 2020), increased fear concerning COVID-19 (Satici, 2020), and the severity of COVID-19 in one’s geographical location (Zhang S. X. et al., 2020).

In order to successfully protect both physical and psychological health during COVID-19, individuals were required to adapt to new circumstances and develop new routines (Bu et al., 2020, p. 2). There is clear evidence of changes to people’s behaviors during the COVID-19 pandemic. Globally, people’s leisure activities were sharply curtailed, with many activities, such as shopping and sport participation, prohibited (Bu et al., 2020, p. 2). People spent considerably more time (than pre-COVID-19) at home, exercised less, and consumed more alcohol (Koopmann et al., 2020; Zheng et al., 2020). In addition, many individuals turned to screen-based activities and consumption of other types of media content (such as listening to music) as a coping resource in response to the stress caused by the pandemic (Zheng et al., 2020). It is this behavior—individuals’ interactions with selected media content (notably, listening to music, playing video/computer games, watching TV/movies/streaming videos, and using social media)—upon which the present study is focused. Although the reasoning behind individuals’ increased use of media during COVID-19 is clear, the extent to which media use during this period predicted life satisfaction is yet to be fully elucidated.

For at least two reasons, the relationship between media use and life satisfaction during COVID-19 is likely to be nuanced and complicated. First, although research points to certain wellbeing and social connection benefits from some media consumption (e.g., Graham and Nikolova, 2013; Doc̆an, 2016), it may also be possible to have “too much of a good thing.” Király et al. (2020), for example, noted that although watching TV, using social media, playing video games, and using the internet are often used to reduce feelings of stress, anxiety, and depression, these are potentially addictive behaviors that may give rise to unhealthy and problematic functioning. With high levels of media exposure, people can experience increased anxiety and stress as well as other unintended negative health consequences such as misplaced help-seeking behaviors (Garfin et al., 2020). Thus, a non-linear relationship may exist between (at least some forms of) media use and life satisfaction. Second, different types of media consumption have been shown to relate in different ways to wellbeing outcomes. For instance, people often listen to music in order to self-regulate their mood (Lonsdale and North, 2011; Boer and Fischer, 2012; Schäfer, 2016; Baltazar et al., 2019; Lonsdale, 2019), reduce negative emotional states (North et al., 2004; Sloboda, 2010), and help relieve or manage everyday stress (Laukka, 2007; Krause et al., 2020). Findings regarding the benefits of watching TV/films and using social media are more mixed (e.g., Frey and Benesch, 2008; Cuñado and Pérez de Gracia, 2012; Arampatzi et al., 2016; Groshek et al., 2018). In general, then, different types of media use may elicit different effects on life satisfaction, and we will explore this possibility in the current study.

### The Present Study

Guided by the possibility of nuanced effects of media use on life satisfaction, we explored relationships between various types of media use and life satisfaction. A repeated assessment research design was utilized to capture relationships at both within- and between-person levels. Specifically, the study aim was to determine whether people who used more media had higher (or lower) life satisfaction, and whether individuals reported higher (or lower) life satisfaction when they reported more media use. The target population for this work was university students, a group for whom the COVID-19 pandemic changed many areas of life. Students were required to adapt to new learning environments, change their peer interactions, cope with financial stressors (e.g., loss of employment), and address concerns about family health (Lyons et al., 2020; Zhao et al., 2020). The psychological well-being of university student cohorts around the world decreased in the early stages of COVID-19 (Idowu et al., 2020), with research showing a negative impact of the pandemic on students’ social connectedness (Lyons et al., 2020), stress, and anxiety (Kobbin et al., 2020; Lyons et al., 2020; Odriozola-González et al., 2020; Savitsky et al., 2020; Son et al., 2020; Sundarasen et al., 2020).

The aim of this study was to investigate whether life satisfaction was related to the use of different types of media among university students during early stages of the COVID-19 pandemic. Previous literature points toward positive relationships between music consumption and wellbeing, and we hypothesized such a relationship at both within- and between-person levels in this study. Due to prior mixed findings with respect to relationships between other types of media consumption and wellbeing, we offered no hypotheses in relation to associations between other types of media use and life satisfaction.

## Materials and Methods

### Participants

A total of 127 individuals participated in the study. All participants were students at a university in Queensland, Australia. Of the sample, 24% identified as male, 74% as female, and 2% as non-binary. Participants were aged 18–53 (*M* = 24.73, *Mdn* = 20, *SD* = 8.96). The majority of the sample (83.47%) were of Australian nationality. A total of 44% of the sample were full-time students, 37% worked part-time, 6% worked full-time, and 12% were unemployed. Participants were recruited using a university participation scheme. As compensation for taking part in the study, students who accessed the study obtained credit toward their coursework. The Human Research Ethics Committee at James Cook University granted ethics approval (Approval number: H8074). Data was collected as a part of a larger study examining Australian students’ experiences of COVID-19, and the present study involved only the data concerning media use and life satisfaction across the study period.

### Design and Procedure

The present study used a repeated measures design. Participants were asked to complete a set of six surveys, each of which was separated by a period of 2 weeks. Participants consented to take part in the study in April, 2020. At this time, the participants had recently begun experiencing the strictest restrictions put in place by the Queensland government, and only virtual means of university attendance was permitted^1^. The questions on the first survey asked participants about their experiences prior to any lifestyle changes due to COVID-19. The final (sixth) survey was completed in mid-July, 2020. The administration of the sixth survey corresponded with both the re-opening of state borders and the resumption of children’s sport locally in the State.

Participants accessed the online questionnaires (hosted using Qualtrics) using direct web links. Individuals read the participant information, gave their informed consent (indicated by clicking “yes” or “no” on the online consent webpage), and created a unique, anonymous code prior to completing the first questionnaire. This code was entered at the start of the subsequent questionnaires, which were completed as a series of webpages. Following completion of the final questionnaire, participants were thanked for their participation and debriefed.

When responding to the first questionnaire, participants were asked to provide demographic information (age, gender, nationality, occupation and country of residence). On each of the six questionnaires, participants responded to questions concerning four media-based leisure activities, namely: listening to music; video/computer gaming; TV/movies/streaming videos; and social media. In particular, they were asked to indicate how often they partook in each of the four activities (noting that responses on the first questionnaire pertained to “prior to any lifestyle changes due to COVID-19”; and responses on the other questionnaires pertained to “in your everyday life over the previous week”). Participants responded using a five-point scale (1 = *never*, 5 = *at least once a day*).

A single item was used to measure participant life satisfaction on each questionnaire (Bu et al., 2020). This item was phrased as, “All things considered, how satisfied have you been with life as a whole this past week?” (or in the case of the first questionnaire, “prior to any lifestyle changes due to COVID-19?”). Participants were asked to use a slider to indicate their response, where 0 = *completely dissatisfied* and 100 = *completely satisfied*. Single-item life satisfaction measures have been shown to perform similarly to other, multi-item measures (Cheung and Lucas, 2014; Atroszko et al., 2017).

### Data Management and Analyses

In total, there were 470 assessments from 127 participants. Most participants completed 6 (*n* = 32, 25%) or 5 (*n* = 26, 20%) surveys, with 18 participants (14%) completing 4, 11 (9%) completing 3, 20 completing 2 (16%), and 20 (16%) completing 1 survey.

Intraclass correlations (ICCs) were used to evaluate how much life satisfaction and media use changed over the six assessments. The hypotheses were tested using multilevel modeling, to account for nesting of data within-person, in the *lme4* (Bates et al., 2015) package of *R* (R Core Team, 2019). To test how life satisfaction and media use changed over time, the study variables were regressed onto time. In *a priori* assumption testing, it was found that change across time in life satisfaction and media use was non-linear; therefore, time was treated as a categorical variable (1–6 assessment time points) in the models with the reference being the first assessment reflecting “everyday life prior to any lifestyle changes due to COVID-19.”

To test between- and within-person associations between life satisfaction and media use, life satisfaction was regressed onto between- and within-person partitions of time spent listening to music, playing video/computer games, watching TV/movies/streaming videos, and using social media. Between-person variables were calculated as each individual’s average value across the six time points; within-person variables were calculated as the deviation from each individual’s average per time point (Shiffman et al., 2008).

## Results

Table 1 depicts study variable descriptive statistics and bivariate correlations. The ICCs revealed that life satisfaction and media use displayed variability at the between- and within-person levels, with using social media and listening to music the most stable over time, and watching TV/movies/streaming videos and life satisfaction the most variable over time. Listening to music was positively associated with watching TV/movies/streaming videos and using social media. Life satisfaction showed no significant associations with any media use variable. These bivariate associations do not account for nesting within-person; therefore, multilevel modeling is needed to appropriately assess between- and within-person associations.

**TABLE 1:** Descriptive statistics and bivariate correlations of life satisfaction, time spent listening to music, playing video/computer games, watching TV/movies/streaming videos, and using social media.

**Variable**	**Mean (*SD*)**	**ICC**	**2**	**3**	**4**	**5**
1. Life satisfaction	66.26 (22.71)	0.51	0.07	0.00	−0.09	0.00
2. Listening to music	4.20 (0.99)	0.64	–	0.02	0.20*	0.22*
3. Playing video/computer games	1.98 (1.29)	0.55		–	0.03	−0.12
4. Watching TV/movies/streaming videos	3.81 (1.02)	0.47			–	0.18*
5. Using social media	4.34 (0.97)	0.69				–

ICC, Intraclass correlation. Pearson bivariate correlations do not account for nesting within-persons across three occasions. *p < 0.05.

### Changes in Life Satisfaction and Media Use

**FIGURE 1:**
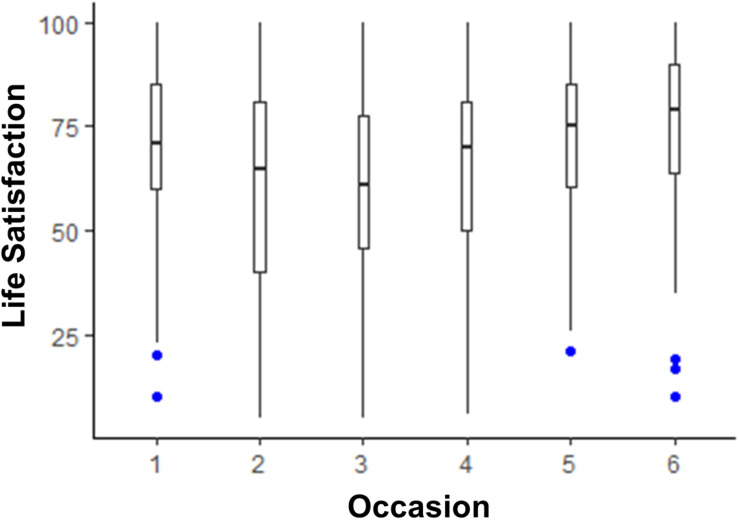
The trend of life satisfaction across six 2-week assessment occasions with the initial assessment reflective of “everyday life prior to any lifestyle changes due to COVID-19.”

**FIGURE 2:**
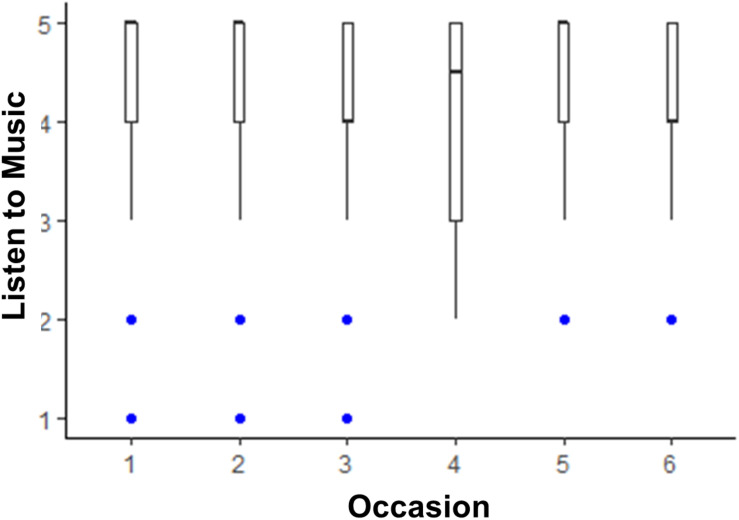
The trend of time spent listening to music across six 2-week assessment occasions with the initial assessment reflective of “everyday life prior to any lifestyle changes due to COVID-19.”

**FIGURE 3:**
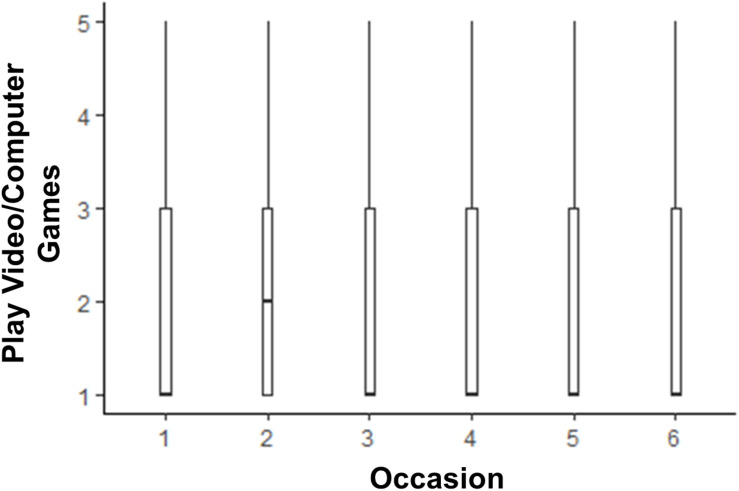
The trend of time spent playing video/computer games across six 2-week assessment occasions with the initial assessment reflective of “everyday life prior to any lifestyle changes due to COVID-19.”

**FIGURE 4:**
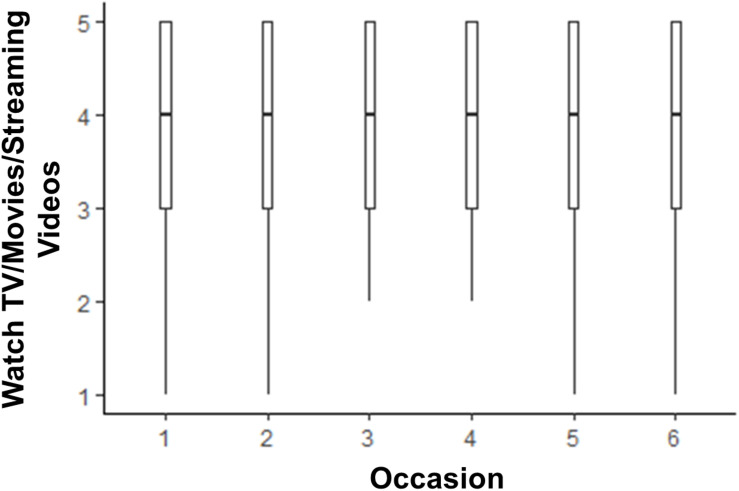
The trend of time spent watching TV/movies/streaming videos across six 2-week assessment occasions with the initial assessment reflective of “everyday life prior to any lifestyle changes due to COVID-19.”

**FIGURE 5:**
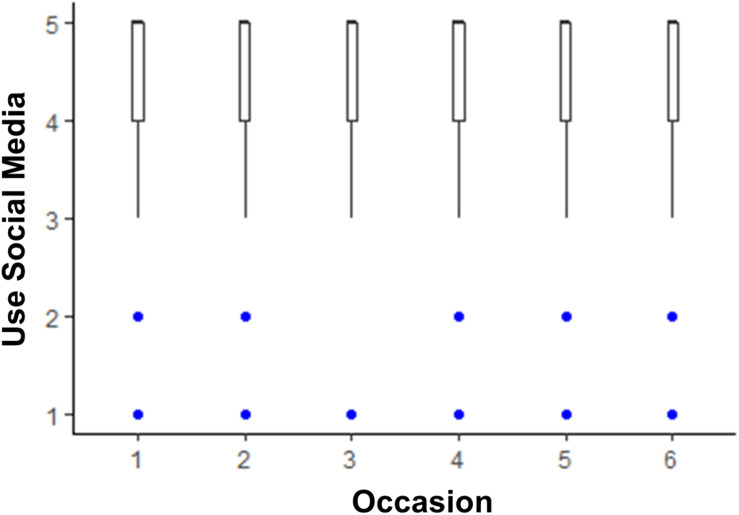
The trend of time spent using social media across six 2-week assessment occasions with the initial assessment reflective of “everyday life prior to any lifestyle changes due to COVID-19.”

The models testing whether life satisfaction and media use changed across the six 2 weeks assessments are shown in Table 2. Life satisfaction scores were lower than pre-COVID levels until time points 5 and 6, when they were no longer statistically significantly lower than pre-COVID. The box plot depicting this trend is shown in Figure 1. There was little change from pre-COVID to during COVID for listening to music, although there was a slight dip in time spent listening to music at time point 3. The box plot depicting listening to music over time is shown in Figure 2. There was an acute increase in video/computer game playing at the time point following the pre-COVID assessment, but this trend dissipated back to a null difference from pre-COVID time for the rest of the time points (see Figure 3). Time spent watching TV/movies/streaming videos was reported more often during time points 2 and 6 compared to pre-COVID, but there were not significant differences between pre-COVID and time points 3–5 (see Figure 4). Time spent using social media was higher during time points 2–4 but dropped back to pre-COVID levels for time points 5 and 6 (see Figure 5).

**TABLE 2:** Multilevel model regression estimates for testing change in life satisfaction and time spent listening to music, playing video/computer games, watching TV/movies/streaming videos, and using social media across 6 two-week assessments with ‘everyday life prior to any lifestyle changes Due to COVID-19’ as the reference comparison.

**Dependent variable**	**Life satisfaction**		**Listening to music**		**Playing video/computer games**		**Watching TV/movies/streaming videos**		**Using social media**	
					
	***b***	**95% CI**	***b***	**95% CI**	***b***	**95% CI**	***b***	**95% CI**	***B***	**95% CI**
Intercept	70.45*	66.61–74.28	4.35*	4.18–4.51	1.94*	1.71–2.16	3.70*	3.52–3.88	4.22*	4.05–4.39
Occasion two	−10.90*	−15.06–−6.73	–0.15	−0.32–0.02	0.29*	0.07–0.51	0.24*	0.03–0.44	0.23*	0.08–0.37
Occasion three	−11.79*	−16.25–−7.33	−0.19*	−0.37—0.01	0.09	−0.14–0.32	0.16	−0.06–0.39	0.23*	0.08–0.37
Occasion four	−7.85*	−12.31–−3.39	–0.14	−0.32–0.04	–0.08	−0.31–0.15	0.21	-0.00–0.43	0.19*	0.03–0.35
Occasion five	–3.29	−8.40–1.83	–0.00	−0.20–0.20	–0.05	−0.31–0.21	0.16	−0.09–0.40	0.11	−0.07–0.29
Occasion six	0.41	−4.43–5.26	–0.11	−0.30–0.08	0.03	−0.21–0.28	0.24*	0.00–0.47	0.13	−0.04–0.30

CI, confidence interval. 474 observations from N = 127, *p < 0.05.

### Life Satisfaction and Media Use

The model testing between- and within-person associations of life satisfaction with media use (Table 3) revealed that there were no significant between-person associations. At the within-person level, however, listening to music was positively associated with life satisfaction, and watching TV/movies/streaming videos was negatively associated with life satisfaction. That is, on occasions when people listened to more music than was usual for them, they reported higher life satisfaction, and on occasions when people watched more TV/movies/streaming videos than was usual for them, they reported lower life satisfaction.

**TABLE 3:** Multilevel model regression estimates for testing between- and within-person associations between life satisfaction and time spent listening to music, playing video/computer games, watching TV/movies/streaming videos, and using social media.

**Dependent variable: life satisfaction**	***b***	**95% confidence interval**
Intercept	72.98*	48.38–97.58
Between-person effects		
Listening to music	0.49	−3.72 to 4.70
Playing video/computer games	–0.63	−3.56 to 2.31
Watching TV/movies/streaming videos	–2.00	−5.99 to 1.98
Using social media	–0.13	-4.18 to 3.91
Within-person effects		
Listening to music	2.97*	0.23–5.71
Playing video/computer games	–0.03	−2.14 to 2.09
Watching TV/movies/streaming videos	−2.53*	−4.83 to −0.24
Using social media	1.59	−1.59 to 4.80

474 observations from N = 127, *p < 0.05.

## Discussion

In the present study, we explored university students’ media use and life satisfaction during COVID-19 restrictions in Australia. Findings reinforced previous work demonstrating positive associations between listening to music and well-being. Consistent with our hypothesis, data showed that life satisfaction was higher than typical levels on occasions when individuals increased their time spent listening to music. We offered no hypotheses in relation to associations between life satisfaction and other types of media use; however, data indicated that when individuals watched more TV/movies/videos, they reported lower life satisfaction. One interpretation of these findings is that people’s media engagement leads to changes in life satisfaction; however, it is important to note that the directionality of these relationships is unclear. For instance, it is possible that people might listen to more music and find that it improves their life satisfaction, yet it is also possible that when people are feeling good about life, they are prone to listening to more music. Consistent with other research undertaken during COVID-19 (e.g., Kamarianos et al., 2020; Lyons et al., 2020; Son et al., 2020), we found high overall engagement with media throughout the assessment period. Listening to music was uniformly popular at the different time points (as demonstrated in Figure 2), whereas other types of media were used most frequently in the early time points (when more government restrictions were in place).

In the COVID-19 context, these findings align with other research considering people’s leisure behaviors during the COVID-19 pandemic. In particular, although the present study did not address directionality, the results align with Bu et al. (2020) findings that reading, hobbies, and listening to music were associated with improvements in mental health and well-being, whereas watching TV and following the news on COVID-19 were associated with declines in mental health and well-being. Moreover, our findings broadly support previous research that has linked TV watching with lower levels of happiness/life satisfaction, while music listening is related to higher life satisfaction (e.g., Frey and Benesch, 2008). While the present data cannot disentangle the reasons for the difference in associations with life satisfaction between music listening and TV watching (streaming), one might speculate as to whether it has to do with the level of user control. Previous research has indicated that listening to music is a relatively active (rather than passive) activity and that greater individual control over what is heard is linked to more positive outcomes (e.g., Krause et al., 2014, 2015). Work on social media use (e.g., Verduyn et al., 2015) has also shown that passive use (rather than active use) is associated with declines in well-being. Thus, it is possible that music listening involves higher levels of individual input than watching TV, which may be associated with positive outcomes. An awareness of the associations between media use and life satisfaction may be useful in informing guidelines around leisure and well-being during COVID-19, as well as during other periods of social lockdown (Tan et al., 2020).

People often interact with media as a coping strategy. Thus, understanding the associations between common media interactions, employed as coping strategies, on life satisfaction has implications for assisting people in modifying their leisure behaviors. This pertains to everyday life, but also is especially important relative to experiences of social isolation and loneliness, prominent during COVID-19. As feelings of loneliness can negatively impact people choosing healthy coping behaviors (Moore and March, 2020), the present findings demonstrate that not all media use aligns with positive well-being outcomes. Despite being unable to establish causation from our findings, it is possible that music listening might be an adaptive coping strategy during times of social isolation. It is interesting to consider this in light of recent work highlighting how listening to music may reduce loneliness and act as a social surrogate (Krause, 2020; Schaäfer and Eerola, 2020; Schaäfer et al., 2020). While individuals seek comfort and company by engaging in both music listening and TV watching, music listening evokes memories and is used to temporarily satisfy needs for social relatedness (Schaäfer and Eerola, 2020). As Schaäfer and Eerola (2020) noted, when people want to connect with specific people, they turn to music rather than TV. Given that the COVID-19 virus affected people’s isolation and interaction, it is possible that even private music listening could convey the presence of other people, thereby acting as a social surrogate. Lyrics of popular music, for instance, typically involve singers speaking about themselves or to “you”—such narration may create “conversation” between singer and listener.

The current findings, in alignment with other work on the benefits of music listening for well-being, support music listening as a potential strategy to improve life satisfaction. Music is widely available, low cost, and enjoyed by a large majority of the population (Schäfer et al., 2013). Taking advantage of these benefits is especially crucial for vulnerable groups, such as students—there is growing evidence that university students report many barriers to seeking help with mental health concerns (Gulliver et al., 2010; Son et al., 2020). It may be beneficial, then, to explore the benefits of music therapy or music listening for students during periods of social isolation and hardship. Phone applications that use music to support mindfulness and well-being may be important to promote during such times. Additional future research might consider how best to embed music listening into existing mental health interventions (de Witte et al., 2020).

### Limitations and Future Directions

The current study is not without limitations. Firstly, it is important to acknowledge that the sample draws on university students and that these students resided in only one area of Australia during the pandemic limiting the study’s generalizability. One strength of the repeated measures design was that participants’ responses were captured over a period of time aligned with changes to COVID-19 restrictions. Although as noted in the section “Materials and Methods,” it was still limited to only one period of COVID-19’s global timeline, which contextualizes the findings. Moreover, it must be acknowledged that the pre-COVID-19 data is based on recall rather than direct experience and that only around half of the sample completed measures at five or six time points. Secondly, the online data collection methods capitalized on self-report, Likert-scale and sliding scale responses. In this way, the data are limited as the method did not capture variations between each assessment period (such that the entire period was not captured) or allow participants to qualify their experiences. Nonetheless, administering the study online was ideal for rapidly responding to COVID-19 with samples from cyber-connected nations, like Australia (van Agteren et al., 2020).

Future research is needed to deepen the level of detail concerning the use of media-based leisure activities as a coping mechanism in everyday life, and during crises like the COVID-19. Data collection methods that permit monitoring of actual media usage, other longitudinal methodologies (e.g., diaries, experience sampling), real-time assessment (e.g., event-contingent assessments) and mixed-methods approaches would facilitate such efforts. Further, the current study did not include measures of participants’ mood, health, or well-being, which might influence people’s leisure and media engagement as well as their level of life satisfaction. Other variables could additionally be considered; for instance, it would be interesting to consider people’s living situation (e.g., the number of people residing in the home) as well as how leisure and media use might relate to social interaction and feelings of both loneliness and isolation. This is especially important given the use of digital technologies can bridge physical distance (Galea et al., 2020), making it likely that greater use of digital technologies will continue given social distancing and lockdown measures are still in place.

Future work could probe relationships between coping mechanisms (and motivations) and stressors. It would be interesting to consider how media use motivations might map onto the perceived functions and consequences of the media use. With such work, it would also be fruitful to further interrogate media preferences, access, and use. For instance, demarcating the use of different technologies to engage in these media-based leisure activities (e.g., listening to music via mobile phone collection vs. streaming music videos on YouTube) may address the role of contextual elements of people’s leisure experiences such as the level of user input and control. In addition, it would be important to consider long-term stress and trauma at later phases in pandemics (Son et al., 2020). Informed by findings concerning time use and well-being, longitudinal research that spans later, “recovery” phases of the pandemic would afford opportunities to capture “both risk and resilience mechanisms” (Dvorsky et al., 2020). This is especially important because the COVID-19 pandemic is still a global concern, and, additionally, we are yet to see the “full aftermath” of both the physical and mental health problems brought on by the virus (Amaya and Melnyk, 2020, p. 7). As Amaya and Melnyk (2020, p. 7) stated, “there is likely to be a tsunami of these issues stemming from the pandemic.” With little doubt that COVID-19 will continue to have a major impact (Dvorsky et al., 2020), this research lends to the broader body of work that works to consider the impact of the COVID-19 pandemic on people’s well-being and life satisfaction.

## Data Availability Statement

The datasets presented in this article are not readily available because ethical approval for this project was granted on the basis that participants would explicitly consent to the possible re-use of their data by the researchers, but ethical clearance was not obtained for the sharing of the collected data. Requests to access the datasets should be directed to AK, Amanda.Krause1@jcu.edu.au.

## Ethics Statement

The studies involving human participants were reviewed and approved by James Cook University (Ethics ID: H8074). The patients/participants provided their written informed consent to participate in this study.

## Author Contributions

AK and JD collaboratively developed the study, gained ethical approval, and conducted participant recruitment. AK oversaw data collection. AR conducted the data analysis, with input from AK, JD, and BJ. AK and AR drafted initial versions of the manuscript, with JD and BJ offering later input. All authors collaborated to approved the final version of the manuscript.

## Conflict of Interest

The authors declare that the research was conducted in the absence of any commercial or financial relationships that could be construed as a potential conflict of interest.

## Footnotes

1 The severity of COVID-19 as well as government-led restrictions in Australia varied between states (Tan et al., 2020; van Agteren et al., 2020). The following information is offered to contextualize participants’ experience of the COVID-19 pandemic with regard to what was happening in Queensland, Australia, at the time of data collection (drawing on information reported by the Australian Broadcasting Corporation, https://www.abc.net.au/news/2020-03-28/coronavirus-timeline-queensland-tracking-spread/12077602). While the Queensland government declared a public health emergency on 29 January 2020, the shutdown of non-essential services and State border closures was announced on 23 March 2020. The Queensland Premier tightened social-distancing and lockdown restrictions with a two-person household rule and banned travel (except for the essential reasons of getting food, medical reasons, work, and exercise) on 30 March 2020. At that time, the student participants experienced a pivot from face-to-face teaching to virtual teaching, which remained in place for the rest of 2020. The first easing of restrictions in Queensland was announced on 26 April 2020, with pubs, clubs, restaurants, and cafes permitted to reopen with restrictions on 16 May 2020. In July 2020, Queensland implemented “stage 3” easing of restrictions with the State border reopening to travelers from other states and territories (excluding Victoria) on 10 July 2020.
